# Role of Serine Proteases in the Regulation of Interleukin-8_77_ during the Development of Bronchopulmonary Dysplasia in Preterm Ventilated Infants

**DOI:** 10.1371/journal.pone.0114524

**Published:** 2014-12-04

**Authors:** Mallinath Chakraborty, Eamon P. McGreal, Andrew Williams, Philip L. Davies, Wendy Powell, Salima Abdulla, Nikolai N. Voitenok, John Hogwood, Elaine Gray, Brad Spiller, Rachel C. Chambers, Sailesh Kotecha

**Affiliations:** 1 Department of Child Health, School of Medicine, Cardiff University, Cardiff, United Kingdom; 2 Centre for Inflammation and Tissue Repair, Rayne Institute, University College London, London, United Kingdom; 3 Fund for Molecular Haematology and Immunology, Moscow, Russia; 4 Division of Haematology, National Institute for Biological Standards and Control, Potters Bar, Hertfordshire, United Kingdom; Helmholtz Zentrum München/Ludwig-Maximilians-University Munich, Germany

## Abstract

**Rationale:**

The chemokine interleukin-8 is implicated in the development of bronchopulmonary dysplasia in preterm infants. The 77-amino acid isoform of interleukin-8 (interleukin-8_77_) is a less potent chemoattractant than other shorter isoforms. Although interleukin-8_77_ is abundant in the preterm circulation, its regulation in the preterm lung is unknown.

**Objectives:**

To study expression and processing of pulmonary interleukin-8_77_ in preterm infants who did and did not develop bronchopulmonary dysplasia.

**Methods:**

Total interleukin-8 and interleukin-8_77_ were measured in bronchoalveolar lavage fluid from preterm infants by immunoassay. Neutrophil serine proteases were used to assess processing. Neutrophil chemotaxis assays and degranulation of neutrophil matrix metalloproteinase-9 were used to assess interleukin-8 function.

**Main Results:**

Peak total interleukin-8 and interleukin-8_77_ concentrations were increased in infants who developed bronchopulmonary dysplasia compared to those who did not. Shorter forms of interleukin-8 predominated in the preterm lung (96.3% No-bronchopulmonary dysplasia vs 97.1% bronchopulmonary dysplasia, p>0.05). Preterm bronchoalveolar lavage fluid significantly converted exogenously added interleukin-8_77_ to shorter isoforms (p<0.001). Conversion was greater in bronchopulmonary dysplasia infants (p<0.05). This conversion was inhibited by α-1 antitrypsin and antithrombin III (p<0.01). Purified neutrophil serine proteases efficiently converted interleukin-8_77_ to shorter isoforms in a time- and dose-dependent fashion; shorter interleukin-8 isoforms were primarily responsible for neutrophil chemotaxis (p<0.001). Conversion by proteinase-3 resulted in significantly increased interleukin-8 activity *in vitro* (p<0.01).

**Conclusions:**

Shorter, potent, isoforms interleukin-8 predominate in the preterm lung, and are increased in infants developing bronchopulmonary dysplasia, due to conversion of interleukin-8_77_ by neutrophil serine proteases and thrombin. Processing of interleukin-8 provides an attractive therapeutic target to prevent development of bronchopulmonary dysplasia.

## Introduction

Persistent lung inflammation, in the form of a poorly resolved neutrophilia, is implicated in the pathogenesis of bronchopulmonary dysplasia (BPD) [1], which is a common disease of preterm infants [2], [3]. Interleukin-8 (IL-8) is a key mediator of lung inflammation in preterm infants [4], attracting polymorphonuclear leucocyte (PMN) to the lungs [1].

IL-8 (CXCL-8) is an early response chemokine, which is a key chemoattractant for PMNs to sites of inflammation. It is synthesised by a variety of cells including alveolar macrophages, endothelial cells, epithelial cells, fibroblasts etc. on induction by inflammatory stimuli. In-vitro, IL-8 seems to be the dominant CXC chemokine [5] produced by alveolar macrophages and accounts for most of the chemotactic activity on PMNs [6]. It has consistently been detected in increased concentration from epithelial lining fluid in lungs of ventilated preterm infants who later develop BPD [4], [7], and has been observed to increase before the peak of inflammatory cell influx [8]. High levels of IL-8 in lung at birth correlate well with increased duration of ventilation in small preterm infants [9]. Results from observational studies on IL-8 in the lungs of preterm infants suggests a key role in persistent neutrophil-driven inflammation, and consequent lung injury, observed in infants developing BPD [10].

IL-8, a member of the CXC [5] family of chemokines, is expressed by a wide variety of cells [11]. Due to variations in the number of amino-acids (aa) at the amino-terminal, several isoforms of IL-8 have been described [12], [13], [14], with the 72 aa isoform (IL-8_72_) being the best characterised. IL-8_72_ is expressed mainly by immune cells [15], [16] while the 77-aa protein, IL-8_77_, is the major isoform expressed by non-immune cells [17], [18]. Functionally, IL-8_72_ and other shorter isoforms are more potent than IL-8_77_ *in vitro* [19], [20], [21], [22] and *in vivo* [23], [24]. Several enzymes can convert IL-8_77_ to shorter isoforms including α-thrombin [19], plasmin [25], matrix metalloprotease-9 (MMP-9) [26] and proteinase-3 (PR3) [27], resulting in significantly increased functional potency [19], [25], [26].

Recently, we reported predominance of IL-8_77_ in the preterm circulation [28]. While total IL-8 has been measured in preterm lungs [29], the concentration of IL-8_77_ or its contribution to the total IL-8 pool is unknown. As significant pulmonary inflammation is observed in infants who develop BPD, we hypothesised that concentration of the potent shorter isoforms of IL-8 would predominate in the preterm lung. Our specific aims were to (a) measure concentration of IL-8_77_ in bronchoalveolar lavage fluid (BALF) of preterm ventilated infants, (b) understand mechanisms of production and post-translational modification of IL-8_77_ in the preterm lungs, and (c) compare differences between infants who develop BPD and those who do not. Our results suggest PR3 and thrombin are key regulators of IL-8_77_ in BALF from preterm ventilated infants developing BPD.

## Materials and Methods

(Additional details are provided in File S1)

### Patient Groups and sample collection

Recruitment of ventilated preterm infants born before 32 weeks gestation, collection and processing of BALF has been previously described [30], [31]. Infants were categorised as: BPD group who required respiratory support or supplemental oxygen (moderate to severe BPD [32]) and No-BPD group who were nursed in air, both at 36 weeks post-conceptional age. Cord blood from term infants, delivered by elective caesarean section, and peripheral venous blood from healthy adult volunteers were collected and processed as described previously [33]. Ethical approval was obtained from the Research Ethics Committee, Cardiff & Vale University Health Board, Cardiff, UK, and prior written informed consent was obtained from parents and healthy adult volunteers.

### ELISAs

Commercial ELISAs were used to measure chemokines (except IL-8_77_) and matrix metalloproteinase-9 (MMP-9) according to the manufacturer's instructions.

Concentration of IL-8_77_ was measured as previously described [34], except samples were detected by the detection-antibody from the commercial IL-8 ELISA described above. Lower limit of sensitivity of the assay was 3.125 pg/ml (Figure S2 in File S1).

PR3 antigen was measured in preterm BALF with an in-house ELISA using a mouse monoclonal anti-PR3 antibody (File S1).

### Conversion of IL-8_77_ by BALF

Exogenously added recombinant human (rh) IL-8_77_ was incubated overnight with buffer, BALF or BALF with protease inhibitors. Samples were collected at 0 hour and 18 hours and stored at -80°C until further analysis.

### Airway Epithelial Cell Culture and Stimulation

Cells were obtained from European Collection of Cell Cultures (ECACC) and grown in 75 cm^3^ flasks (Corning Life Sciences, Amsterdam, The Netherlands) containing DMEM media with L-glutamine for A549 cells or F12-K media with L-glutamine for BEAS-2B cells (both media from HyClone, Cramlington, UK) and 5% heat-inactivated (HI) foetal calf serum (FCS, Sigma-Aldrich Company Ltd., Gillingham, UK), incubated in a humidified incubator at 37°C and 5% CO_2_. Cells were allowed to grow to 80-90% confluence in a monolayer before being sub-cultured, using 0.25% Trypsin EDTA (Lonza, Slough, UK). Cell viability was checked by 0.4% Trypan Blue exclusion (Thermo Scientific, Loughborough, UK).

Human small airway epithelial cells (SAEC, Lonza CC-2547, Slough, UK) were cultured in 25 cm^2^ flasks (Corning Life Sciences, Amsterdam, The Netherlands) in SAEC basal media containing growth supplements (Clonetics SAGM BulletKit CC-3118) according to Lonza's guidelines.

Epithelial cells were seeded into 6-well plates at a density of 3×10^5^ cells/ml and allowed to adhere for more than 12 hours prior to washing with saline and further culture in serum-free media (A549 and BEAS-2B) for six hours (SAECs were grown in defined serum-free medium). Cells were washed again in saline prior to stimulation with IL-1β (5 ng/ml for A549 and BEAS-2B cells and 1 ng/ml for SAEC) in duplicate for 18 hours at 37°C and 5% CO_2_; supernatants were collected after incubation and immediately frozen at −80°C until further analysis.

### PMN and monocyte purification and stimulation

PMNs and mononuclear cells from term cord blood and from healthy adult human volunteers were cultured in RPMI 1640 medium with 10% heat-inactivated foetal calf serum and stimulated with lipopolysaccharide (LPS) in duplicate for 18 hours at 37°C and 5% CO_2_. Supernatants were stored at −80°C until further analysis.

### Conversion of IL-8_77_ by purified proteases

Purified human neutrophil elastase (NE), cathepsin-G (CG) and PR3 were incubated with rhIL-8_77_ at 37°C in buffer. Samples were collected at specified time-points from 0 min to 24 hours and the reaction was immediately stopped by adding alpha-1 antitrypsin (AAT); samples were stored at -80°C until further analysis by ELISA.

### Neutrophil Degranulation Assay

Purified PMNs from healthy adult human volunteers were exposed to cytochalasin B for 15 minutes before stimulation by N-Formyl-Met-Leu-Phe (fMLP), rhIL-8_72_ or rhIL-8_77_ controls at 10 nM each, or samples collected from conversion experiments with purified proteases. After incubation for 30 min at 37°C, samples were centrifuged at 200 g for 2 min and supernatants stored at -80°C until further analysis.

### Neutrophil Chemotaxis Assay

Chemotaxis of isolated human neutrophils (2×10^5^ per well) was measured in response to infant BALF, with or without 10 µg/ml of anti-total human IL-8 neutralising antibody or anti-human IL-8_77_ neutralising antibody. Migrated cells were counted (cells/ml) with a haemocytometer after 60 min incubation.

### Thrombin activity assay

Thrombin activity in preterm BALF was measured using a specific substrate S2238 (total activity). Background activity was measured by inhibiting the activity with hirudin and specific activity was calculated after subtracting background from total activity.

### Statistical Analysis

Statistical analysis was performed on GraphPad Prism version 5.03 for Windows (GraphPad Software, San Diego, CA, USA). Non-normally distributed clinical data are reported as medians and inter-quartile range (IQR); data from all other experiments, which were normally distributed, are reported as means and standard error of mean (SEM). Data from BALF incubations and time-course experiments with IL-8_77_ and purified proteases are expressed as relative fold change from 0 hour. Comparison of data between two groups was performed by Mann-Whitney U test (clinical data) and two-tailed unpaired t-test (all other data). Data between multiple groups was compared by one-way ANOVA with post-hoc multiple-comparison tests as appropriate (details mentioned in figure legends). Data from time-course and dose-response experiments were compared by two-way ANOVA with Bonferroni correction. Categorical data was compared by using Fisher's exact test. Correlations were assessed by using Spearman's rank correlation test. p<0.05 was considered significant.

## Results

### Clinical Data

Clinical characteristics of the two groups are shown in table 1. Thirty-eight samples were analysed from 22 infants (11 No-BPD and 11 BPD). The number of ventilation-days (p = 0.004) and peak fractional oxygen requirement (FiO2; p = 0.002) were significantly higher in the BPD infants but all other characteristics were similar between the two groups.

**Table 1:** Patient and clinical characteristics.

	No-BPD	BPD (moderate to severe)	p
Total infants	11	11	
Total samples	13	25	
Gestational age (weeks)*	28^+3^ (27–29^+4^)	27^+5^ (25^+4^–29^+2^)	0.45
Birth weight (g)*	1120 (960–1250)	905 (798.8–1120)	0.14
Prolonged rupture of membranes (>24 hours)^∧^	1 (10)	2 (22)	1.0
Antenatal steroids (≥24 hours)^∧^	9 (82)	9 (82)	1.0
Caesarean delivery^∧^	5 (45)	6 (55)	1.0
Exogenous surfactant replacement^∧^	11 (100)	11 (100)	1.0
Mechanical ventilation-days*	1.0 (1.0–1.75)	6.5 (5.0–15.5)	0.004
Peak PIP*	17.0 (15.75–21.5)	22.0 (20.0–25.0)	0.07
Peak FiO2*	21.0 (21.0–30.5)	41.0 (32.5–70.5)	0.002
Patent ductus arteriosus^∧^	5 (45)	8 (73)	0.39
Peak C-Reactive Protein (CRP)*	4.0 (1.5–14.5)	18.0 (6.5–82.0)	0.08
Lung Infection (16sRNA)^∧^	2 (18)	4 (36)	0.64

*Values shown are medians (inter-quartile range) with superscript numbers indicating days in addition to gestation in weeks; ^∧^ values shown are numbers of infants (percentage of total in group).

### Expression of IL-8 isoforms and chemokines

**Figure 1:**
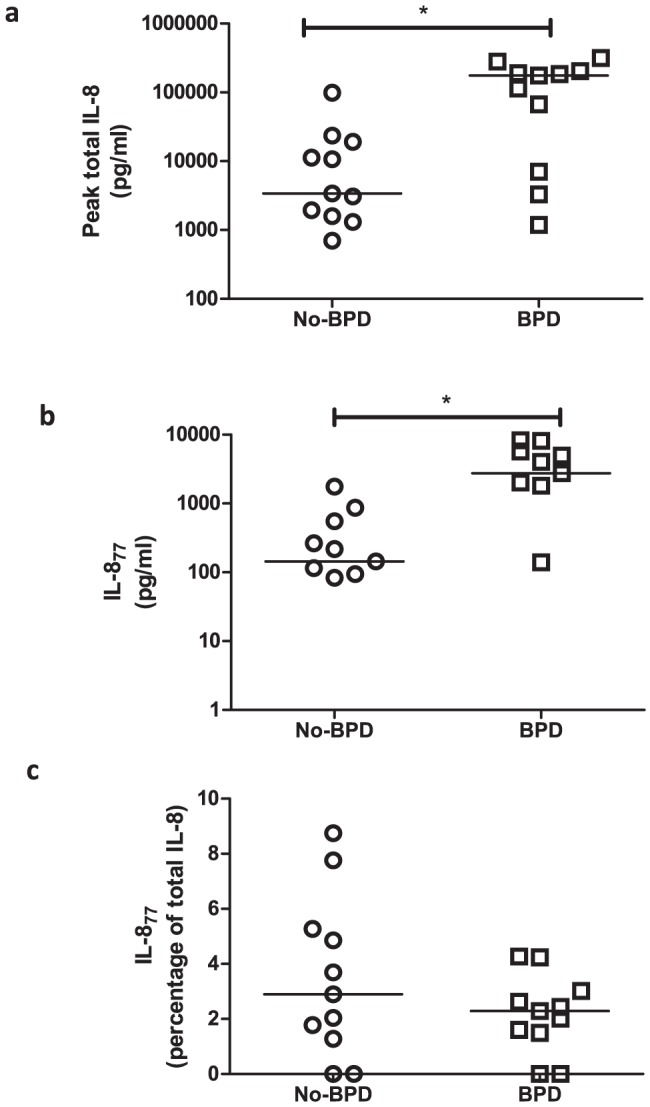
Expression of IL-8 and IL-8_77_ in preterm BALF. (a) Peak concentration of total IL-8, (b) corresponding IL-8_77_ and (c) proportion of IL-8_77_, expressed as a percentage of total IL-8, in preterm BALF from infants in the No-BPD group (circles, n = 11) and BPD group (squares, n = 11). Groups are represented on the x-axis while concentration of each antigen (pg/ml, log scale) or proportion (as a percentage) is represented on the y-axis. Each point represents a single infant and bars are at medians. (* = p<0.05)

IL-8 was detected in all 38 samples but IL-8_77_ was below the detection limit in three BPD and two No-BPD samples. Peak total IL-8 concentration (median No-BPD 3401 pg/ml, inter-quartile range 1587–19080 pg/ml *vs* BPD 175600 pg/ml, 7064–202700 pg/ml; p = 0.01, Figure 1a) and corresponding IL-8_77_ (No-BPD 144.3 pg/ml, 83.6–552.6 pg/ml *vs* BPD 2753 pg/ml, 140.1–5668 pg/ml; p = 0.03, Figure 1b) concentration in BALF were significantly higher in the BPD infants compared to No-BPD infants. However, IL-8_77_ was a minor proportion of the total IL-8 in all of the samples, and there was no significant difference between the two groups (IL-8_77_ in No-BPD median 2.9%, 1.3–5.3% *vs* BPD 2.3%, 1.5–3.0%; p = 0.39, Figure 1c); significant correlation was observed between total IL-8 expression and corresponding IL-8_77_ concentration in preterm BALF (n = 22, Spearman r = 0.94, p<0.0001; Figure S3 in File S1). Thus, concentration of the shorter isoforms of IL-8 predominated in BALF from preterm ventilated infants.

The majority of IL-8 in preterm circulation is reported to be IL-8_77_, in contrast to term infants or adult circulation [28]. However, no significant correlation was observed between gestation and IL-8_77_ concentration (n = 13, r = −0.47, p = 0.1; Figure S4a in File S1) or the proportion (percentage) of IL-8_77_ (n = 13, r = −0.14, p = 0.65; Figure S4b in File S1) in preterm BALF. Only samples from day 1 of life were included in this analysis to reflect in-utero concentration.

No significant correlation was found between the peak inspiratory pressure (PIP) or peak fractional oxygen requirement (FiO2) with IL-8 expression in either of the two groups of infants. When all infants are analysed together, there was moderate correlation between PIP and IL-8 expression in BALF (r = 0.6, p = 0.01).

Since other chemokines can attract neutrophils by binding the same receptors as IL-8, we measured peak concentration of growth regulated oncogene-α (GRO-α, CXCL1), GRO-β (CXCL2) and epithelial neutrophil-activating protein-78 (ENA-78, CXCL5). While GRO-α was significantly increased in the BPD infants (p<0.05), there were no significant differences in concentration of GRO-β or ENA-78 between the two groups (figure S1 in File S1).

### IL-8_77_ Expression by Cells *in vitro*

**Figure 2:**
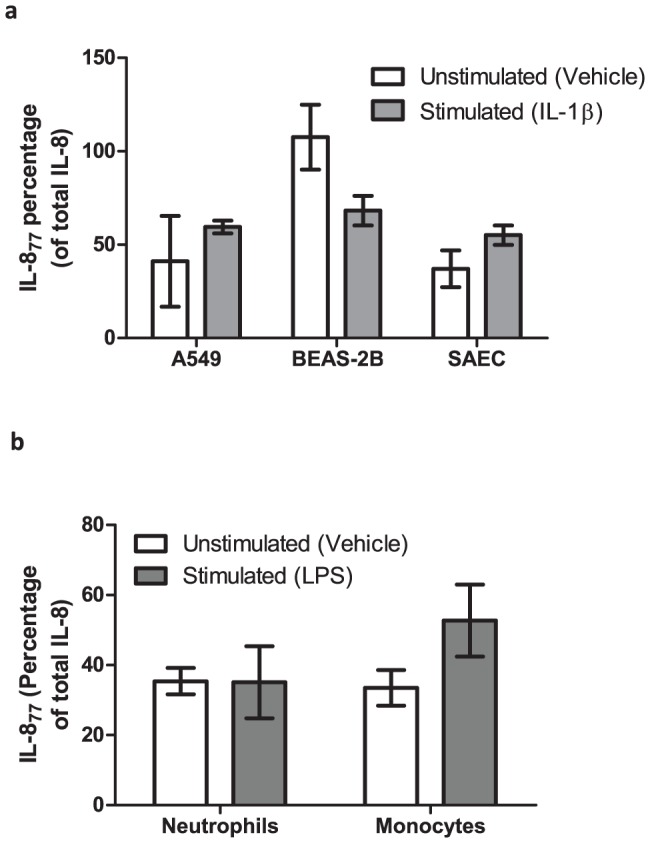
Expression of IL-8_77_ from cultured cells. Proportion of IL-8_77_, expressed as a percentage of total IL-8, from un-stimulated (open bars) and stimulated (shaded bars) (a) airway epithelial cells and (b) term cord-blood neutrophils and monocytes. Cells-types are represented on the x-axis while percentage of IL-8_77_ is represented on the y-axis. Bars are means (± SEM) of three independent cell-culture experiments, each conducted in duplicate. All supernatants were separately measured by ELISA in duplicate.

We next looked at expression of IL-8_77_ in two adult lung cell-lines A549 (alveolar epithelial cells) and BEAS-2B (bronchial epithelial cells) and also in primary adult small-airway epithelial cells (SAEC), as well as in cord blood PMNs and monocytes from term infants delivered by elective caesarean section. Although unstimulated A549 cells expressed negligible IL-8, this increased 29-fold on stimulation, with 60% of it being IL-8_77_ (Figure 2a and S5a in File S1). Unstimulated BEAS-2B cells expressed all of their IL-8 as IL-8_77_; on stimulation, there was an 89-fold increase in expression of total IL-8, of which around 66% was IL-8_77_ (Figure 2a and S5a in File S1). SAECs expressed 25% of their IL-8 as IL-8_77_ in the absence of any stimulation; this increased to 56% (Figure 2a and S5a in File S1) on stimulation, which also resulted in a 10-fold increase in expression of total IL-8. Term cord-blood PMNs expressed approximately 34% of their IL-8 as IL-8_77_ with or without stimulation (Figure 2b and S5b in File S1). Unstimulated and stimulated cord monocytes expressed 33% and 53% of IL-8 as IL-8_77_ respectively (Figure 2b and S5c in File S1). These data suggest that a significant proportion of IL-8 expressed by resident and infiltrating cells in the lungs is the IL-8_77_ form.

### Convertase Activity of BALF

**Figure 3:**
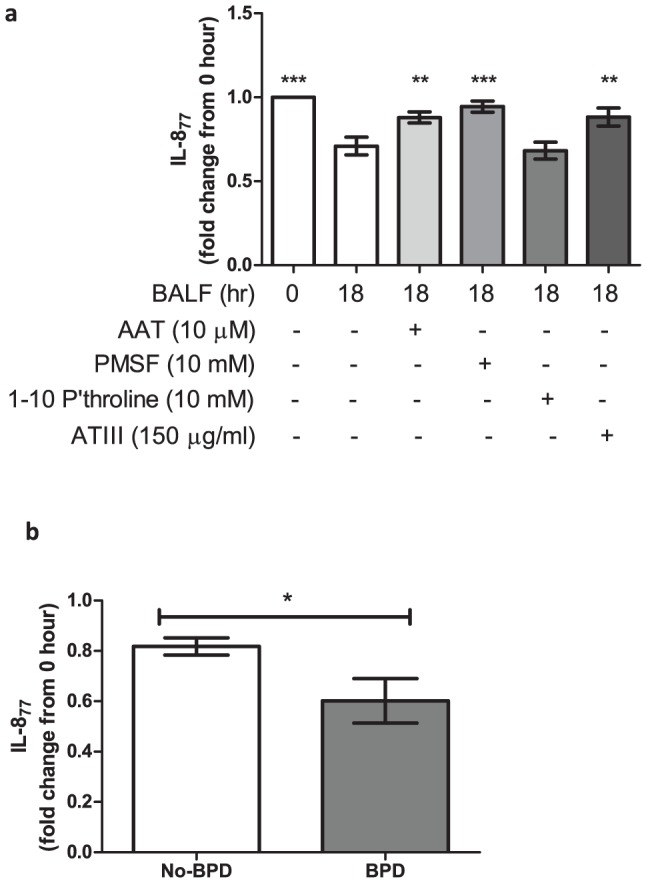
Convertase activity of preterm BALF. (a) Concentration of IL-8_77_ detected by ELISA at 0 hours and after incubation in different conditions for 18 hours. Conditions are detailed on the x-axis and fold change of IL-8_77_ (compared to concentration at “0-hour”) on the y-axis. All bars are at means (± SEM). Conditions were compared by repeated measures ANOVA with Dunnets correction, comparing against a control column (BALF at 18 hour, n = 18). (b) Concentration of IL-8_77_ detected by ELISA at 18 hours in No-BPD (open bar, n = 9) and BPD (shaded bar, n = 9) infants. Infant groups are detailed on the x-axis and concentration of IL-8_77_ (fold change from “0-hour”) on the y-axis. All bars are at means (± SEM). (* = p<0.05, ** = p<0.01, *** = p<0.001)

We have shown that a minority of the IL-8 in preterm BALF is expressed as the IL-8_77_ form, although it is the predominant form expressed by stimulated lung cells. As several proteases are known to convert IL-8_77_ to shorter isoforms [19], [25], [26], [27], we investigated processing of IL-8_77_ by protease activity of preterm BALF with appropriate controls (Figure S6 in File S1). On incubating BALF with exogenously added rhIL-8_77_ (10 ng/ml = 1.1 nM), recovery of this isoform after an 18 hour incubation was reduced by 30% compared to initial concentration (p<0.001, Figure 3a). To explore the mechanism of this conversion, a number of specific inhibitors were used: alpha-1 antitrypsin (AAT) for neutrophil serine proteases, phenylmethylsulfonyl fluoride (PMSF) for serine proteases, 1-10 phenanthroline for metalloproteases and antithrombin-III (ATIII) for thrombin. While both AAT (p<0.01) and PMSF (p<0.001) resulted in significant inhibition of conversion of IL-8_77_ by preterm BALF, no similar inhibition was observed with 1-10 phenanthroline, suggesting neutrophil serine proteases, and not metalloproteases, were responsible for this conversion (Figure 3a). The inhibition of convertase activity by ATIII (p<0.01) suggested that thrombin and other coagulation proteases were active within the preterm lung. There was significantly increased convertase activity in BPD infants (leading to decreased recovery of IL-8_77_) compared to No-BPD (p = 0.03, Figure 3b). To look at possible differential effect of proteases in the two groups, we have analysed "protection" from conversion by AAT, ATIII and PMSF for BPD and No-BPD infants separately. There were no statistically significant differences for any of the antiproteases between the two groups; however, protection in the No-BPD group was high regardless of the anti-protease used while for the BPD group, maximum protection was achieved by PMSF (has broader anti-protease effect than AAT or ATIII individually). AAT provided greater protection than ATIII in the BPD group.

### Conversion of IL-8_77_ by purified neutrophil proteases

**Figure 4:**
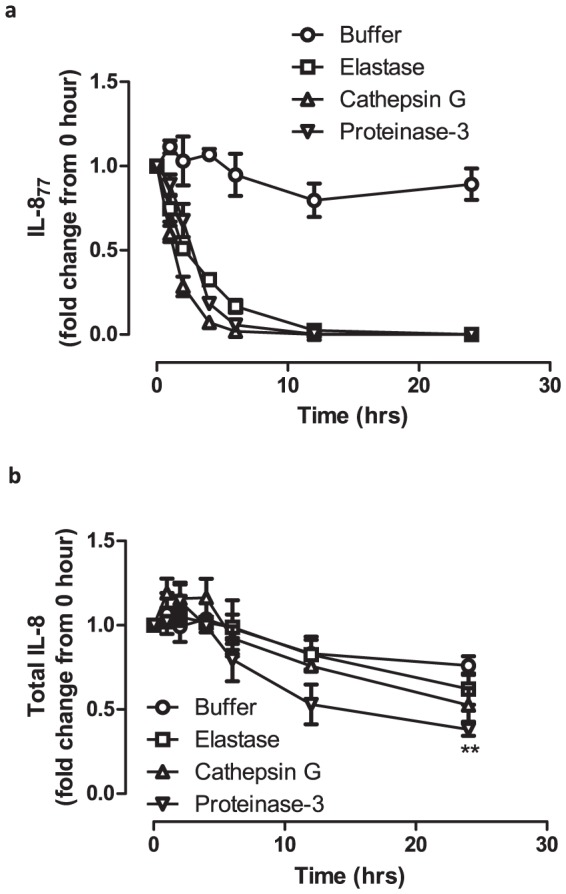
Time-course of conversion of IL-8_77_ by purified proteases. (a) Fold change in concentration of IL-8_77_ and (b) total IL-8 on incubation with by purified neutrophil proteases at different time-points. Time (hours) is represented on the x-axis while the fold-change in concentration (compared to concentration at “0-hour”) is represented on the y-axis. Points plotted are means (± SEM) of three independent experiments, each measured by ELISA in duplicate. Differences in concentration was compared by two-way ANOVA with Bonferroni's correction. (** = p<0.01; p-values for figure 4a are mentioned in the text)

Our data suggested that active neutrophil and coagulation cascade serine proteases in BALF can significantly convert exogenously added IL-8_77_. The role of thrombin and plasmin has been studied previously [19], [25]. We next proceeded to study the pattern of this conversion by the three neutrophil serine proteases NE, CG and PR3 individually. All three proteases showed significant convertase activity when incubated with rhIL-8_77_, when compared with buffer control at all time-points (Figure 4a) and varying doses of proteases (Figure S7 in File S1). This was time-dependent and the majority of this conversion took place by 6 hours (17% of IL-8_77_ remaining with NE, 2% with CG, 6% with PR3; Figure 4a). Among the three serine proteases, CG was the most potent; percentage of remaining IL-8_77_ was significantly lower when compared with NE after 2 hours (p<0.05) and 4 hours (p<0.05) of incubation; and also on comparison with PR3 after 1 hour (p<0.01) and 2 hours (p<0.001) of incubation. The percentage of remaining IL-8_77_ was not significantly different between NE and PR3 at any time-point. IL-8_77_ was converted to shorter isoforms of IL-8, which were detectable by immunoassay for total IL-8 (Figure 4b). However, after incubation with proteases for>12 hours, there was a trend towards further degradation of IL-8 to peptides undetectable by ELISA. This was most evident with PR3 where total IL-8 detected (38% of starting concentration) was significantly lower when compared with buffer control (76% of starting concentration) at 24 hours (p<0.01). Such an effect has been reported previously with an excess of NE [35] resulting in loss of functional activity.

### Functional activity of IL-8_77_ after incubation with proteases

**Figure 5:**
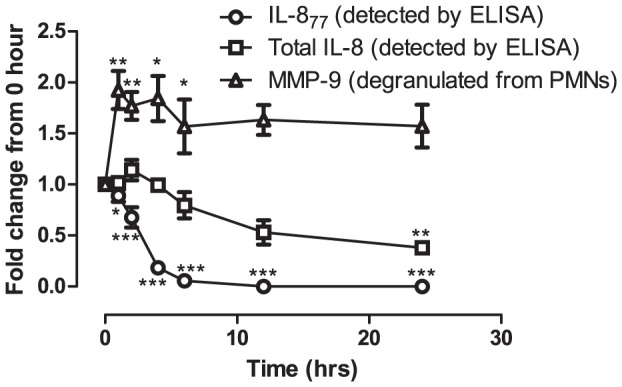
Modulation by proteinase-3. Summary of processing of rhIL-8_77_ by purified proteinase-3 showing recovery of total IL-8 (squares) and IL-8_77_ (circles) and release of MMP-9 from neutrophils in response to the products of conversion (triangles). Time (hours) is represented on the x-axis while IL-8/MMP-9 (fold change from 0-hour values) is represented on the y-axis. Points plotted are means (± SEM) of three independent experiments, each measured by ELISA in duplicate. Statistical differences in concentration compared to buffer-control at each time-point was tested by 2-way ANOVA with Bonferroni's correction. (* = p<0.05, ** = p<0.01, *** = p<0.001).

We have shown that all three neutrophil serine proteases efficiently convert IL-8_77_ to shorter isoforms over time. Next, we proceeded to test the functional activity of the converted products in a neutrophil degranulation assay (figure S8 in File S1). We hypothesised that if IL-8_77_ was converted to functional shorter isoforms, then it would be reflected in increased functional potency of the products. Although NE and CG efficiently converted IL-8_77_ (Figure 4a), no significant differences in functional activity were observed between the products from NE or CG, compared to buffer-control, at any time-point (Figure S9 in File S1). In contrast, there was a significant increase in potency in the products after incubation with PR3 (Figure 5) after 1 and 2 hours (p<0.01 compared to buffer control) and 3 and 4 hours (p<0.05 compared to buffer control). This suggests that although all the three serine proteases can convert IL-8_77_, only conversion by PR3 results in a significant increase in functional potency suggesting a key regulatory role for this enzyme towards IL-8_77_ in the preterm lung.

### PR3 antigen expression and thrombin activity in preterm BALF

**Figure 6:**
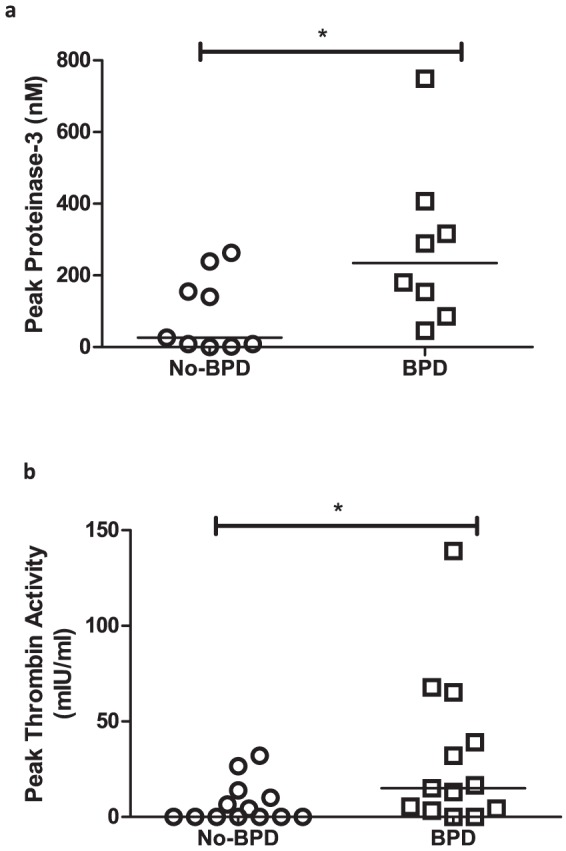
Proteinase-3 antigen and thrombin activity in preterm BALF. Peak (a) proteinase-3 expression (nM) and (b) thrombin activity (mIU/ml) in BALF from No-BPD (circles, n = 9 for proteinase-3 antigen and n = 13 for thrombin activity) and BPD (squares, n = 8 for proteinase-3 antigen and n = 13 for thrombin activity) infants. Groups are represented on the x-axis while concentration of each antigen (nM) or activity (mIU/ml) is represented on the y-axis. Points plotted are from individual infants and bars are at medians. (* = p<0.05)

Both peak PR3 antigen expression (p = 0.03, Figure 6a) and peak thrombin activity (p = 0.03, Figure 6b) were significantly higher in BALF from BPD infants compared to No-BPD infants when measured in an *in vitro* assay. Thrombin activity was measured in BALF from a wider cohort of preterm ventilated infants (same epoch and collected and processed identically), due to limited BALF availability.

### Neutrophil chemotaxis to preterm BALF

**Figure 7:**
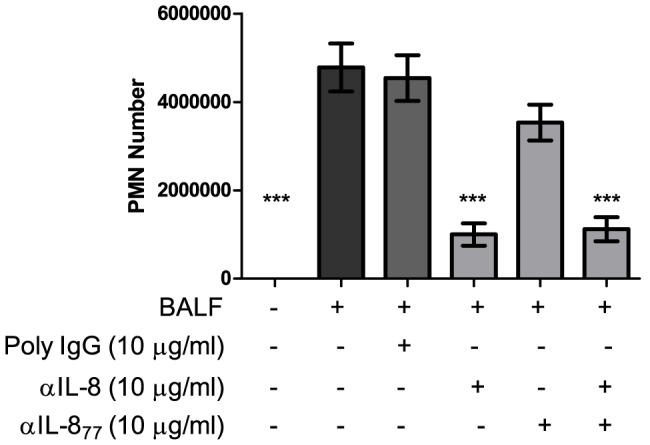
Neutrophil chemotaxis to preterm BALF. Chemotaxis of adult human neutrophils to pooled BALF from BPD infants after incubation for 60 min. Conditions are detailed on the x-axis while concentration of neutrophils (cells/ml) are expressed on the y-axis. Bars are at means (± SEM) of three independent experiments, each done in triplicate. Conditions were compared by one-way ANOVA with Dunnet's correction, comparing against a control column (BALF only). (*** = p<0.001).

To establish the importance of IL-8 as a chemokine in preterm BALF, chemotaxis of purified adult human neutrophils to pooled BALF from BPD infants was assessed. Inhibition of total IL-8, but not IL-8_77_, resulted in significant reduction of neutrophil chemotaxis (figure 7) suggesting that shorter forms of IL-8 were primarily responsible for the effect.

## Discussion

This is the first report of IL-8_77_ expression in the lungs of preterm infants. Although total IL-8 and IL-8_77_ concentration were significantly increased in infants who developed BPD, the majority of the IL-8 measured in preterm BALF seemed to be the more potent shorter isoforms. Resident and infiltrating cells in the lung express a significant proportion of IL-8 as the 77 aa isoform. We have demonstrated a significant capacity of neutrophil serine proteases and thrombin in the preterm BALF to convert IL-8_77_ to its shorter isoforms. Specifically, conversion by PR3 resulted in shorter isoforms with increased functional activity. The activity could, however, be inhibited by appropriate anti-proteases.

Persistent, neutrophil-driven inflammation has been strongly implicated in the pathogenesis of BPD with IL-8 being a key chemokine involved in the process. Our results are in keeping with previous data that IL-8 concentration is significantly increased in the lungs of infants developing BPD [4]. Although mechanical ventilation and oxygen toxicity are known to contribute to lung inflammation in preterm infants [10], in our cohort, IL-8 expression was not significantly correlated with PIP or with FiO2 in either of the two groups of infants when examined individually. IL-8_77_ was detected at lower concentration, which agrees with our hypothesis that the shorter isoforms constitute the majority of the IL-8 concentration in preterm BALF, and is in keeping with persistent neutrophil influx into the lungs. However, this is in contrast to the higher proportion of circulating IL-8_77_ in preterm infants (>60%) as reported recently [28]. Two possibilities, to explain the low concentration of IL-8_77_ in preterm BALF, were reduced expression or post-translational modification. Pulmonary epithelial cell lines are known to express IL-8 on stimulation [36], [37]; however, expression of IL-8_77_ has not been described before. We have shown for the first time that IL-8_77_ constitutes a significant proportion of the total IL-8 secreted by a variety of airway cell-lines and primary small airway cells, both unstimulated and on stimulation. Other abundant cell types in the preterm lung include neutrophils and cells of the monocytes/macrophage lineage. Previously, IL-8_77_ expression has been reported to range from 17–38% of total IL-8 from adult monocytes [14] and mixed mononuclear cells [13], and about 60% of total IL-8 from PMNs [27]. We have described the expression of IL-8_77_ from term neonatal cord-blood PMNs and monocytes. Our results are in general agreement with adult data published previously. Taken together, these data suggest that both resident and infiltrating cells in the lung could express significant quantities of IL-8_77_, especially on stimulation.

As resident and infiltrating cells in the lungs express significant proportion of IL-8_77_, the low concentration of IL-8_77_ in the preterm lungs is likely to be due to post-translational modification. Indeed, BALF from all infants significantly converted exogenously added IL-8_77_ after 18 hours of incubation, which was more pronounced in the BPD infants. This was mediated mainly by neutrophil serine proteases and thrombin. As we have shown previously [38], excess bovine serum albumin (BSA) has no inhibitory effect on the activity of the neutrophil serine proteases, leading us to conclude that this is unlikely to be a non-specific inhibition. AAT itself can bind to IL-8 (substrate sequestration) to reduce its bioavailability [39], a property which could have influenced our results. We have demonstrated inhibition of IL-8_77_ cleavage by PMSF, suggesting protease inhibition is the mechanism resulting in this effect. We believe further studies will need to be conducted to confirm the precise mechanism *in vivo*. The role of proteolytic enzymes in the pathogenesis of BPD is still being debated. Early studies in preterm infants have suggested the involvement of neutrophil proteases in contributing to inflammation and lung injury [40], [41], and several proteases, including NE and metallo-proteinases [42], [43], [44] have been implicated in the pathogenesis of BPD. However, data from other studies have been less clear on the relationship [45]. Experimental and adult data seem to support the hypothesis that uncontrolled proteases activity can contribute to lung injury [46], [47], as observed in lung autopsy specimens of infants with BPD [48]. We have recently shown in the preterm lung that increased expression of these proteases can be episodic, related to infective episodes, and is associated with the development of BPD [31]. Apart from the direct effect of proteases, post-translational modification of chemokines [49] can also influence local immune function and have an impact on tissue injury. This effect is particularly important for IL-8, as cleavage by proteases may result in isoforms with enhanced functional activity.

As shown previously, PR3 converts IL-8_77_ to IL-8_70_, but NE and CG were not involved in the conversion process [27]. We have shown that all three of the main serine proteases in PMNs can convert IL-8_77_ to shorter isoforms. This is effective both over time and over a dose range. CG was the most potent and NE the least potent. Interestingly, with longer incubation periods, there was a decrease in the amount of total IL-8 recovered, most evident with PR3. This suggests the existence of several potential cleavage sites for the enzymes on the IL-8 molecule, which are distal from the NH_2_-terminal. NE has previously been reported to abolish IL-8 activity by a similar process [35], although the authors did not find a similar effect with CG (PR3 was not studied). In our study, all three PMN serine proteases reduced IL-8 recovery at the concentrations tested. PR3 initially increased functional potency of IL-8_77_, but over time IL-8 was cleaved into smaller peptides that were undetectable by ELISA; similar results were obtained on incubating IL-8_72_ with PR3 (Figure S10 in File S1). Taken together, these results suggest that PR3 may be a key modulator of IL-8 function in the preterm lungs, which seems to be a key chemoattractant in preterm BPD BALF (figure 7). This could have significant implications for lung inflammation leading to BPD, and further studies need to be conducted to confirm the clinical effects of this modulation *in vivo*.

Cross-talk between inflammation and the coagulation cascade has previously been reported [50], [51]. To our knowledge, only one previous study has reported thrombin activity in BALF from preterm ventilated infants [52]; surprisingly, they found lower activity in BPD infants compared to No-BPD infants on days 2 and 4 of life. In a recent *in vitro* model, activation of the coagulation cascade by BPD-BALF was shown to promote fibrotic responses [53]. Activation of the clotting cascade has been shown to contribute to fibrotic lung diseases [54], [55] where thrombin is part of the final common coagulation pathway. Our analysis suggests that thrombin is involved in modulating lung inflammation in ventilated preterm infants by post-translational modification of IL-8, thus attracting increased numbers of neutrophils into the lungs expressing inflammatory mediators and serine proteases. Recently, inhibition of neutrophil serine proteases was shown to improve lung growth in a murine model of BPD [56]. Taken together our data suggest that inhibition of specific proteases could regulate IL-8 activity and modulate pulmonary inflammation in preterm infants at risk of BPD; an *in vivo* study would be needed to confirm this hypothesis.

Although IL-8 has consistently been reported at higher concentration in the lungs of infants developing BPD, there may be other chemokines involved in neutrophil chemotaxis via stimulation of the IL-8 cognate receptors [57]. The concentration of BALF GRO-α was significantly higher in BPD infants, but those of GRO-β and ENA-78 were comparable in the BPD and No-BPD groups, suggesting that these chemokines may not be as dominant as IL-8 during neutrophil migration. Furthermore, BALF from BPD infants was highly chemotactic for neutrophils. This chemotactic response was significantly inhibited by neutralising total IL-8, but not IL-8_77_, thereby suggesting that the shorter IL-8 isoforms are more potent neutrophil chemoattractants involved in the pathogenesis of BPD.

IL-8_77_ seems to be the predominant isoform of IL-8 found in the systemic circulation of preterm infants [28]. Moreover, preterm plasma has significantly lower convertase activity for IL-8_77_ compared to term or adult plasma [28]. Since chemotatic potency of IL-8_77_ is significantly lower than IL-8_72_, this unique pattern of expression of the IL-8 isoforms in the preterm circulation could be designed to attenuate inflammation *in utero*. It is possible that IL-8_77_ may have other non-inflammatory developmental roles in the foetus. However, in a localised inflammatory milieu like the preterm ventilated lungs, IL-8 seems to have a predominantly inflammatory role resulting in the persistent influx, and activation, of neutrophils into the lungs. The low concentration of IL-8_77_ in preterm BALF, possibly due to conversion into the more potent shorter isoforms, supports this hypothesis. We speculate that inhibition of this conversion could potentially shift the balance towards attenuation of lung inflammation and injury. This hypothesis needs to be tested *in vivo*.

We acknowledge certain limitations of this study. The airway cell-lines and primary cells used in our experimental models are all from adult populations and may not reflect conditions in the preterm lung accurately. The transcriptional activity of preterm lung cells is not known; however, they are known to be less mature than term cells [1]. We have also used term cord-blood cells to look at expression of the chemokine. We believe that the future use of appropriate models may enable us to overcome some of these difficulties. We have measured PR3 antigen concentration; it is possible that all of it may not be active. We also acknowledge possible differences in *in vitro* and *in vivo* activity, and it is very likely that the latter is more efficient; this needs to be addressed in future studies. Our study was undertaken with the aim of defining the extent to which IL-8_77_ was expressed in the preterm lung given that a previous study reported IL-8_77_ to be the dominant form of IL-8 in circulation in preterm infants. Having demonstrated that IL-8_77_ was not dominant in airway lavage fluid, we undertook a series of experiments to understand why this might be the case. We acknowledge that there may well be additional mechanisms at play which are beyond the scope of this study.

In conclusion, our data show that in the lungs of ventilated preterm infants, the majority of IL-8 expression seems to be the shorter (more potent) isoforms; this is also supported by the pattern of cellular infiltration into the lungs. The concentration of IL-8_77_ is low in preterm BALF, although it has been previously detected as a considerable proportion of total IL-8 in the preterm circulation. PR3 and thrombin are key modulators of IL-8 in the preterm lung – targeting of processing of IL-8 provides an attractive therapeutic target to prevent pulmonary inflammation in infants at risk of developing BPD.

## Supporting Information

File S1 **Supporting information.** Figure S1, Chemokines in preterm BALF. Peak concentration of (a) CXCL1, (b) CXCL2 and (c) CXCL5 in preterm BALF. Groups are represented on the x-axis while concentration of chemokines (pg/ml) are represented on the y-axis. Each point represents peak value from a single infant and bars are at medians (* = p<0.05). Figure S2, Modification of IL-8_77_ ELISA. Antigens IL-8_77_ (open circles) and IL-8_72_ (open squares) were captured by (a) N11 and (b) BD capture antibody. Both were detected by the BD detection antibody. Concentration of the antigens is represented on the x-axis (pg/ml) while absorbance at 450 nm (corrected at 570 nm) is represented on the y-axis. Points represent means (± SEM) of three independent experiments. Values were compared by two-way ANOVA with Boferroni's post-test (*** = p<0.001). Figure S3, Expression of IL-8 and IL-8_77_. Concentration of peak total IL-8 (pg/ml) and corresponding concentration of IL-8_77_ (pg/ml) are plotted on the x- and y-axis respectively. Each point represents a single infant and correlation was tested by calculating Spearman's coefficient. Figure S4, Gestation and IL-8_77_ expression. Correlation of gestation at birth with (a) concentration of IL-8_77_ and (b) percentage of IL-8 expressed as IL-8_77_, in preterm BALF. Birth gestation of infants (weeks) is plotted on the x-axis while first day IL-8_77_ concentration (pg/ml) or proportion of total IL-8 (percentage) in preterm BALF is plotted on the y-axis. Each point represents a single infant and correlation was tested by calculating Spearman's coefficient. Figure S5, Expression of IL-8_77_ from cells. Concentration (pg/ml) of total IL-8 (open bars) and IL-8_77_ (shaded bars) from (a) airway epithelial cells and term cord-blood (b) PMNs & (c) monocytes, both unstimulated (U) and when stimulated (S) with IL-1β (airway epithelial cells) or LPS (cord-blood cells). Cell-lines and incubation conditions are represented on the x-axis while concentration (pg/ml) is on the y-axis. Bars are at means (± SEM) of at least three separate experiments. Figure S6, Panel of controls. Concentration of IL-8_77_ detected by ELISA at 0 hours and after incubation in different conditions for 18 hours. Conditions are detailed on the x-axis and concentration of IL-8_77_ (pg/ml) on the y-axis. Open bars represent buffer controls, grey bar represent BALF samples at 0 hour and black bar represents BALF samples after 18 hours incubation (n = 18). All bars are at means (± SEM). Conditions were compared by one-way ANOVA with Dunnets post-hoc test comparing against a control column (BALF at 0 hour) (*** = p<0.001). Figure S7, Protease dose-response. Conversion of IL-8_77_ by purified human neutrophil (a) elastase, (b) cathepsin-G and (c) proteinase 3 at varying concentration. Ratio of enzyme: substrate is represented on the x-axis while the fold-change of total IL-8 (circles) and IL-8_77_ (squares) from original concentration is represented on the y-axis. Points plotted are means (± SEM) of three independent experiments. Concentration of IL-8_77_ is compared to corresponding concentration of total IL-8 by two-way ANOVA with Bonferroni's post-test (* = p<0.05, ** = p<0.01, *** = p<0.001). Figure S8, Establishing the neutrophil degranulation assay. MMP-9 expressed from neutrophils after degranulation by control conditions (open bars), rhIL-8_72_ (grey bars) and rhIL-8_77_ (black bars). Details of conditions are represented on the x-axis while expression of MMP-9 is represented on the y-axis (expressed as a fold change compared to the positive control). Bars are at means (± SEM) of three independent experiments. Difference in means was compared by one-way ANOVA with Tukey's post-test (comparing all pairs of columns). */***  =  significant compared to negative control; ##  =  significant compared to rhIL-8_72_ at a concentration of 10^−8^ M (* = p<0.05, *** = p<0.001, ##  = p<0.01). Figure S9, Functional activity of IL-8 isoforms. Summary of processing of rhIL-8_77_ by (a) buffer, (b) purified elastase and, (c) purified cathepsin G showing concentration of recovered total IL-8 (squares), IL-8_77_ (circles) and MMP-9 from degranulation of neutrophils (triangles) by the products of conversion. Time (hours) is represented on the x-axis while IL-8/MMP-9 (fold change from 0-hour values) is represented on the y-axis. Points plotted are means (± SEM) of three independent experiments. Statistical differences in concentration compared to buffer-control at each time-point was tested by 2-way ANOVA with Bonferroni's post-test. (*** = p<0.001). Figure S10, Processing of rhIL-8_72_ by purified proteinase-3. Recovery of total IL-8 (expressed as a fold change from 0-hour) on incubation with buffer (circles) and proteinase-3 (squares) over 24 hours. Time (hours) is represented on the x-axis while fold-change in concentration (compared to concentration at “0-hour”) is represented on the y-axis. Points plotted are means (± SEM) of three independent experiments. Differences in concentration was tested by two-way ANOVA with Bonferroni's post-test (*** = p<0.001).
(DOC)
