# Supplementary material for: Role of Serine Proteases in the Regulation of Interleukin-877 during the Development of Bronchopulmonary Dysplasia in Preterm Ventilated Infants
Source: PLoS One. 2014 Dec 4;9(12):e114524. doi: 10.1371/journal.pone.0114524 (PMC4256433; doi:10.1371/journal.pone.0114524)
Supplement: File S1 — Supporting information. Figure S1, Chemokines in preterm BALF. Peak concentration of (a) CXCL1, (b) CXCL2 and (c) CXCL5 in preterm BALF. Groups are represented on the x-axis while concentration of chemokines (pg/ml) are represented on the y-axis. Each point represents peak value from a single infant and bars are at medians (* = p<0.05). Figure S2, Modification of IL-877 ELISA. Antigens IL-877 (open circles) and IL-872 (open squares) were captured by (a) N11 and (b) BD capture antibody. Both were detected by the BD detection antibody. Concentration of the antigens is represented on the x-axis (pg/ml) while absorbance at 450 nm (corrected at 570 nm) is represented on the y-axis. Points represent means (± SEM) of three independent experiments. Values were compared by two-way ANOVA with Boferroni's post-test (*** = p<0.001). Figure S3, Expression of IL-8 and IL-877. Concentration of peak total IL-8 (pg/ml) and corresponding concentration of IL-877 (pg/ml) are plotted on the x- and y-axis respectively. Each point represents a single infant and correlation was tested by calculating Spearman's coefficient. Figure S4, Gestation and IL-877 expression. Correlation of gestation at birth with (a) concentration of IL-877 and (b) percentage of IL-8 expressed as IL-877, in preterm BALF. Birth gestation of infants (weeks) is plotted on the x-axis while first day IL-877 concentration (pg/ml) or proportion of total IL-8 (percentage) in preterm BALF is plotted on the y-axis. Each point represents a single infant and correlation was tested by calculating Spearman's coefficient. Figure S5, Expression of IL-877 from cells. Concentration (pg/ml) of total IL-8 (open bars) and IL-877 (shaded bars) from (a) airway epithelial cells and term cord-blood (b) PMNs & (c) monocytes, both unstimulated (U) and when stimulated (S) with IL-1β (airway epithelial cells) or LPS (cord-blood cells). Cell-lines and incubation conditions are represented on the x-axis while concentration (pg/ml) is on the y-ax [file pone.0114524.s001.doc]

**Role of Serine Proteases in Regulation of Interleukin-8_77_ during the development of Bronchopulmonary Dysplasia in Preterm Ventilated Infants**

Mallinath Chakraborty, Eamon P McGreal, Andrew Williams, Philip L Davies, Wendy Powell, Salima Abdulla, Nikolai N Voitenok, John Hogwood, Elaine Gray, Brad Spiller, Rachel C Chambers, Sailesh Kotecha

**Supplementary Information**

**Materials and Methods**

*Patient Groups*

Ventilated preterm infants born before 32 weeks gestation were recruited, after obtaining written parental consent, from the Regional Neonatal Unit at University Hospital of Wales in Cardiff, UK. Term infants born by elective Caesarean section for maternal reasons (repeat section for previous section, breech presentation at term) and healthy adults were recruited after obtaining written consent from parents and donors respectively. Preterm infants with significant hypoxia, known congenital abnormalities, abnormal morphology or who were extremely unwell (not expected to survive) were excluded from the study. Prior ethical approval for all sample collection was obtained from the Research Ethics Committee. Preterm infants were categorised as: bronchopulmonary dysplasia (BPD) group who required respiratory support or supplemental oxygen at 36 weeks corrected gestational age (moderate to severe BPD) and No-BPD group who were free of respiratory support at the same time-point.

*BALF Collection*

Non-bronchoscopic broncho-alveolar lavage (BAL henceforth) was performed on ventilated infants (daily in the first week of life and twice-weekly thereafter) as previously described . Briefly, infants were disconnected from the ventilator and, with their head turned to the left, an appropriate size suction catheter was passed down the endo-tracheal tube until resistance was felt. 1 ml/kg of warmed 0.9% saline was instilled down the tube and was immediately suctioned out by connecting the upper end of the suction tube to a water trap using wall suction pressure of 8-12 kPa. This was repeated once more and the total volume of fluid instilled and recovered was recorded. The recovered fluid was pooled and placed on ice before being transported to the lab. In the lab, a haemocytometer count of total cells was done from the pooled fluid before it was centrifuged at 4ºC at 1000 g for 10 minutes. The supernatant was removed, aliquoted out to smaller volumes (25µl) and frozen at -80ºC until further use.

*IL-8, GRO-α, GRO-β and ENA-78 ELISA*

Total IL-8 (OptEIA Set for Human IL-8, BD Biosciences, San Diego, CA; lower limit of detection 3.125 pg/ml), GRO-α & ENA-78 (both Duoset, R&D Systems Europe Limited, Abingdon, UK; 31.25 & 15.625 pg/ml respectively) and GRO-β (Abnova, Taipei City, Taiwan; 8 pg/ml) was measured in samples by commercial ELISA according to manufacturer’s recommendations. All samples were freshly thawed-out before the assay.

*IL-8_77_* *ELISA*

Concentration of IL-8_77_ was measured by the method published previously with one modification. NUNC Maxisorp 96-well plates (Fisher Scientific, Loughborough, UK) were coated with 100 μl/well of 5 μg/ml capture antibody N11 (stock 1.3 mg/ml) and incubated overnight at 4°C. Wells were washed with 200 μl wash buffer (x 3) for IL-8_77_ ELISA (phosphate buffered saline + 0.05% Tween-20 + 300 mM NaCl) and blocked with 200 μl of assay diluent (IL-8_77_ wash buffer + 0.5% protease-free bovine serum albumin) for 1 hour at room temperature. The wash step was repeated as before prior to adding 100 μl of standard, BALF or cell-culture supernatant in duplicate. Top standard of 200 pg/ml was prepared from recombinant human IL-8_77_ (Peprotech EC Ltd, London, UK; stock concentration 10 μg/ml) and a seven point standard curve using 2-fold serial dilution was prepared. Assay diluent was used as the negative control. Following a 2 hour incubation and wash step (x 3), 100 μl of the working detector (detection antibody from BD OptEIA human IL-8 ELISA kit and streptavidin-HRPO, each diluted 1 in 250 in assay diluent) was added to each well. After a 1 hour incubation, the wells were washed (x 5) and 100 μl of 3,3',5,5'-Tetramethylbenzidine (eBiosciences, Hatfield, UK) was added. Once the standards had developed, 50 μl of 2 N sulphuric acid was added to stop the reaction. Optical density of each well was determined using a microplate reader (Dynex Technologies, Worthing, UK) at 450 nm with wavelength correction at 570 nm. The assay range was from 3.125 pg/ml to 200 pg/ml.

*Matrix Metalloproteinase-9 (MMP-9) ELISA*

Total matrix metalloproteases-9 was measured in supernatants by commercial ELISA (R&D Systems Europe Limited, Abingdon, UK) as per manufacturer’s recommendations. Lower limit of detection of the assay was 31.25 pg/ml.

*Proteinase-3 ELISA*

Mouse monoclonal anti human proteinase 3 antibody (Hycult Biotechnology, Netherlands) was immobilised onto 96-well plates prior to blocking with 5% BSA in PBS containing 0.5% Tween-20 (blocking buffer). Samples and standards were diluted in blocking buffer and incubated with the coated plates for 1 h. Purified proteinase-3 (Athens Research and Technology, GA, USA) was diluted from 0-250ng/ml for the standard curve. Unbound protein was washed away with PBS prior to detection with rabbit polyclonal anti-proteinase-3 antiserum (Eurogentec, Belgium) and peroxidase-conjugated donkey anti-rabbit immunoglobulin (Jackson Immuno Research Laboratories, Newmarket, UK). Plates were developed with OPD-EASY (Acros Organics, Belgium), and the reaction stopped with 2N H_2_SO_4_ before reading at 490 nm.

*Conversion of IL-8_77_ by BALF*

Conversion of IL-8_77_ to shorter isoforms by BALF was assessed by incubating equal volumes of recombinant human IL-8_77_ (Peprotech EC Ltd, London, UK) at a concentration of 30 ng/ml with buffer (Tris buffered saline with bovine serum albumin: TBS BSA) containing 50 mM Tris (Fisher Scientific, Loughborough, UK) + 154 mM NaCl (Fisher Scientific UK Ltd, Loughborough, UK) + 0.5% protease-free BSA pH 7.4, BALF or BALF with the protease inhibitors alpha-1 antitrypsin (AAT 10 μM, Talecris Plasma Resources, NC, USA) phenylmethylsulfonyl fluoride (PMSF 10 mM, Sigma Aldrich Company Ltd., Dorset, England), 1-10 phenanthroline (10 mM, Sigma Aldrich Company Ltd., Dorset, England) or anti-thrombin III (ATIII 150 μg/ml, Merck Chemicals Limited, Nottingham, UK). BALF was pre-incubated with protease inhibitors or buffer for 30 minutes before adding IL-8_77_; final concentration of IL-8_77_ at the beginning of the incubation period was 10 ng/ml (1.1 nM). A sample was collected at 0 hour and immediately frozen at -80°C. Rest of the samples were incubated overnight for 18 hours and then frozen at -80°C until analysis. The concentration of IL-8_77_ recovered at the end of the incubation period was measured by specific ELISA (as above). The possibility of conversion of IL-8_77_ during incubation in the ELISA was considered and a protease inhibitor cocktail (Sigma Aldrich Company Ltd., Dorset, England) was used at a concentration of 1 in 1000 in the dilution buffer to inhibit any protease activity. Before data analysis, IL-8_77_ concentration in the original sample was subtracted from the concentration recovered after the assay.

*Airway Epithelial Cell Culture and Stimulation*

Cells were obtained from European Collection of Cell Cultures (ECACC) and grown in 75 cm^3^ flasks (Corning Life Sciences, Amsterdam, The Netherlands) containing DMEM media with L-glutamine for A549 cells or F12-K media with L-glutamine for BEAS-2B cells (both media from HyClone, Cramlington, UK) and 5% heat-inactivated (HI) foetal calf serum (FCS, Sigma-Aldrich Company Ltd., Gillingham, UK), incubated in a humidified incubator at 37°C and 5% CO_2_. Cells were allowed to grow to 80-90% confluence in a monolayer before being subcultured using 0.25% Trypsin EDTA (Lonza, Slough, UK). Cell viability was checked by 0.4% Trypan Blue exclusion (Thermo Scientific, Loughborough, UK).

Human small airway epithelial cells (SAEC, Lonza CC-2547, Slough, UK) were cultured in 25 cm^2^ flasks (Corning Life Sciences, Amsterdam, The Netherlands) in SAEC basal media containing growth supplements (Clonetics SAGM BulletKit CC-3118) according to Lonza’s guidelines.

Epithelial cells were seeded into 6-well plates at a density of 3 x 10^5^ cells/ml and allowed to adhere for more than 12 hours prior to washing with saline and further culture in serum-free media (A549 and BEAS-2B) for six hours (SAECs were grown in defined serum-free medium). Cells were washed again in saline prior to stimulation with IL-1β (5 ng/ml for A549 and BEAS-2B cells and 1 ng/ml for SAEC) for 18 hours at 37°C and 5% CO_2_; supernatants were collected after incubation and immediately frozen at -80°C until further analysis.

*PMN and Monocyte Purification and Stimulation*

Purification of PMNs and mononuclear cells from term cord blood and from healthy adult human volunteers was achieved by centrifuging through a discontinuous Percoll (Sigma Aldrich Company Ltd., Dorset, England) gradient as described previously . In summary, after the baby was delivered and cord clamped & cut, the placenta was slowly delivered into a bag. Blood was collected from the vessels in the cord attached to the placenta. 17 ml of blood was collected into a syringe containing 3 ml of 3.8% sodium citrate and immediately processed. For adult volunteers, 18 ml of blood was collected into a syringe containing 2 ml of 3.8% sodium citrate and immediately processed similar to cord blood. Anti-coagulated blood was centrifuged at 450 g for 20 minutes to separate cells from the plasma. The plasma was centrifuged at 1300 g for 20 minutes to obtain platelet poor plasma (PPP), part of which was aliquoted and frozen at -80°C for future use. The cell layer (from above) after separation from plasma was further processed. 3 ml of 6% Dextran solution (from Leuconostoc mesenteroides, average mol wt 425,000 to 575,000 D, Sigma Aldrich Company Ltd., Dorset, England) was added per 20 ml of blood and original volume was reconstituted with 0.9% (w/v) NaCl. Cells were mixed well with dextran by manual rolling and incubated in a water bath at 37°C for 30-45 minutes. Two layers of cells were visible after this step: an upper leukocyte-rich layer and a lower RBC layer with a clear border of separation. The leukocyte layer was carefully aspirated and centrifuged at 200 g for 6 minutes; the pellet at the end of this step was re-suspended in 2 ml of autologous PPP (as before).

Two Percoll gradients were prepared as below:

51% layer – 1.02 ml 90% Percoll + 0.98 ml PPP

42% layer – 0.84 ml 90% Percoll + 1.16 ml PPP

The 51% layer was added to bottom of a 15ml conical tube and carefully overlaid with the 42% layer followed by the leukocyte layer, so as to avoid mixing. The 15 ml tube with the three separate layers was centrifuged at 350 g for 13 minutes without braking. At the end of this step, three layers, two bands and a pellet were visible:

a top layer of plasma

a band of mononuclear cells (MNC)

42% Percoll layer

a band of polymorphonuclear leukocytes (PMN)

51% Percoll layer

RBC pellet at the bottom.

The MNC and PMN bands were carefully and separately aspirated with a 1 ml pipette and re-suspended in 10 ml of Hank’s Balanced Salt Solution (HBSS) without calcium (Ca), magnesium (Mg) or phenol red (Lonza, Slough, UK).

PMNs were washed twice in media and re-suspended in RPMI 1640 (Lonza, Slough, UK) with 10% HI FCS (for cell stimulation experiments) or HBSS with Ca (for degranulation experiments), to adjust their concentration to 5 X 10^6^ cells/ml. 200 μl of cell suspension was added into each well of a 24-well plate (1 X 10^6^ cells/well) and stimulated with 10 ng/ml of LPS for 18 hours. Supernatants were collected at the end of incubation and frozen at -80°C until further analysis.

Mononuclear cell fraction was washed twice in media and re-suspended in RPMI 1640 with 10% HI FCS to a final concentration of 2.5 X 10^6^ cells/ml. 400 μl of cell suspension was pipeted into each well of a 24-well plate (1 X 10^6^ cells/well) and incubated for 30 minutes at 37°C and 5% CO2. Non-adherent cells were washed off by vigorous washing with media and the remaining cells were stimulated with 1 ng/ml of LPS for 18 hours. Supernatants were collected at the end of incubation and frozen at -80°C until further analysis.

*Conversion of IL-8_77_ by purified proteases*

The purified human neutrophil serine proteases elastase (HNE), cathepsin-G (CG) & proteinase-3 (Pr-3) (Athens Research and Technology, Athens, GA USA) were assessed for their ability to convert IL-8_77_ to shorter isoforms. For time-course experiments, 0.6 μM (5.34 μg/ml) of rhIL-8_77_ was incubated with 6.0 μM of protease (enzyme:substrate=10:1) at 37°C (final concentration of substrate 0.3 μM and of enzyme 3.0 μM) in TBS BSA. 20 μl of sample was collected at indicated time-points from 0 minutes to 24 hours and was mixed with 280 μl of plasma derived alpha-1 antitrypsin at 2.14 μM (final concentration of proteases 0.2 μM and of AAT 2.0 μM) to stop the reaction and then frozen at -80°C until further analysis. All samples were analysed for total IL-8 and IL-8_77_ concentrations by ELISA as above.

*Neutrophil Degranulation Assay*

PMNs were purified from healthy adult human volunteers as described previously and their concentration was adjusted to 5 X 10^6^ cells/ml in HBSS containing calcium and magnesium (Lonza, Slough, UK). 200 μL of cells (1 X10^6^ cells) were pipeted into individual test-tubes (Elkay Laboratory Products Limited, Basingstoke, UK). Cells were exposed to buffer (HBSS) or 5 μg/ml of cytochalasin B (Sigma Aldrich Company Ltd., Dorset, England) for 15 minutes before being stimulated by N-Formyl-Met-Leu-Phe (fMLP, Sigma Aldrich Company Ltd., Dorset, England) at 1.0 μM, rhIL-8_72_ (Peprotech EC Ltd, London, UK) or rhIL-8_77_ controls at 100 & 10 nM each, or samples collected at different timepoints from conversion experiments as described before. All samples were equally diluted to contain 10 nM of total IL-8. Samples were incubated with cells for 30 mins in a water-bath at 37°C. At the end of incubation, tubes were centrifuged at 200g for 2 minutes and supernatants and stored at -80°C until further analysis.

*Thrombin Activity Assay*

Thrombin activity was measured by a single point estimation against a calibration curve using the 2^nd^ International Standard for Thrombin (01/580, NIBSC, South Mimms, UK) in a microtitre plate, with colour developed from a thrombin substrate, S2238 (Instrumentation Laboratories, Warrington, UK). To exclude the activity of any additional material present in the samples that could cleave the substrate, a highly specific inhibitor to thrombin, hirudin (Refludan, Pharmion, UK), was used to provide a background level of activity. Thrombin levels were calculated with this background activity excluded.

*Neutrophil Chemotaxis Assay*

Adult human neutrophils were purified over a dual Histopaque gradient (Histopaque 1119, Histopaque 1077, Sigma Aldrich Company Ltd., Dorset, England). Cell count and purity were assessed by microscopy. ChemoTX plates (Neuro Probe, Gathersburg, USA) were used throughout (3 µm pores in a 96-well plate) employing 2 × 10^5^ neutrophils per well. Chemotaxis of isolated human neutrophils was measured in response to infant BAL fluid, with or without 10 µg/ml anti-human IL-8 (total IL-8) neutralising antibody (R&D Systems Europe Limited, Abingdon, UK) or anti- human IL-8_77_ neutralising antibody (Ebioscence, Hatfield, UK). BAL fluid was incubated for 15 min with each neutralising antibody prior to the addition of 2 × 10^5^ neutrophils per well. Migrated cells were counted (cells/ml) with a haemocytometer after 60 min incubation.

**Supplementary Results**

*Measurement of neutrophil chemokines*

Although IL-8 has been consistently reported at higher concentration in the lungs of infants developing BPD, several other chemokines are involved in neutrophil chemotaxis utilising the same cognate receptors as IL-8. We measured the concentration of three other chemokines: GRO-α (CXCL1), GRO-β (CXCL2) and ENA-78 (CXCL5) in preterm BALF. Due to limited sample volume from infants in the study cohort, these were measured from a wider cohort of preterm ventilated infants belonging to the same epoch and collected & processed identically.

As shown in figure S1, peak concentration of GRO-α was significantly higher in the BPD group of infants (p < 0.05). However, there were no statistically significant differences in concentration of GRO-β or ENA-78 between the BPD and No-BPD groups.

a b

c

Figure S1: Chemokines in preterm BALF. Peak concentration of (a) CXCL1, (b) CXCL2 and (c) CXCL5 in preterm BALF. Groups are represented on the x-axis while concentration of chemokines (pg/ml) are represented on the y-axis. Each point represents peak value from a single infant and bars are at medians. (* = p < 0.05)

*IL-8_77_ ELISA*

Using the capture & detection antibody pair for the IL-8_77_ ELISA as described before , the concentration of IL-8_77_ in several BALF samples was found to be below the limit of detection of the assay (10pg/ml) after dilution. In an order to increase sensitivity of the assay, the detection antibody from the BD OptEIA human IL-8 kit was used in the ELISA. Assays were set up with all possible combinations of capture and detection antibodies (Figure 1). The N11 coating antibody was specific for IL-8_77_ within the range of concentrations used in the ELISA and did not bind IL-8_72_ (fig S2a). The BD OptEIA capture antibody was non-specific for both isoforms (detects total IL-8 , fig S2b). Detection antibody from the BD OptEIA human IL-8 ELISA kit detected both isoforms equally well; it also increased the sensitivity of the IL-8_77_ ELISA to a lower limit of 3.125pg/ml.

a b

Figure S2: Modification of IL-8_77_ ELISA. Antigens IL-8_77_ (open circles) and IL-8_72_ (open squares) were captured by (a) N11 and (b) BD capture antibody. Both were detected by the BD detection antibody. Concentration of the antigens is represented on the x-axis (pg/ml) while absorbance at 450 nm (corrected at 570 nm) is represented on the y-axis. Points represent means (± SEM) of three independent experiments. Values were compared by two-way ANOVA with Boferroni's post-test. (*** = p < 0.001)

*Expression of IL-8 and IL-8_77_*

There was significant correlation between the concentrations of peak total IL-8 and corresponding IL-8_77_ (r = 0.94, p < 0.0001, fig S3).

Figure S3: Expression of IL-8 and IL-8_77_. Concentration of peak total IL-8 (pg/ml) and corresponding concentration of IL-8_77_ (pg/ml) are plotted on the x- and y-axis respectively. Each point represents a single infant and correlation was tested by calculating Spearman’s coefficient.

*Gestation and IL-8_77_*

The majority of IL-8 in preterm circulation consists of IL-8_77_, in contrast to term or adult circulation . Thus, it seems that with maturity of the foetus, the expression of IL-8 isoforms in circulation changes. However, in preterm BALF, no significant correlation was observed between either the concentration of (figure S4a) or the proportion of (figure S4b) IL-8_77_ in the preterm lungs with the birth-gestation of infants. For this analysis, only day 1 samples were chosen to reflect in-utero concentrations as closely as possible; 13 such samples were available for analysis.

a b

Figure S4: Gestation and IL-8_77_ expression. Correlation of gestation at birth with (a) concentration of IL-8_77_  and (b) percentage of IL-8 expressed as IL-8_77_, in preterm BALF. Birth gestation of infants (weeks) is plotted on the x-axis while first day IL-8_77_ concentration (pg/ml) or proportion of total IL-8 (percentage) in preterm BALF is plotted on the y-axis. Each point represents a single infant and correlation was tested by calculating Spearman’s coefficient.

*Expression of IL-8_77_ from cells in vitro*

Although unstimulated A549 cells expressed negligible IL-8 (mean concentration 19 pg/ml), this increased 29-fold on stimulation (548 pg/ml, fig S5a). Unstimulated BEAS-2B cells expressed all of their IL-8 as IL-8_77_ (35 pg/ml); on stimulation, there was an 89-fold increase in expression of total IL-8 (3138 pg/ml). SAECs expressed 25% of their IL-8 as IL-8_77_ in the absence of any stimulation; this increased to 56% on stimulation which also resulted in a 10-fold increase in expression of total IL-8 (596 pg/ml to 6037 pg/ml). Term cord-blood PMNs showed a 14-fold increase (106 pg/ml to 1435 pg/ml, fig S5b) and monocytes showed a 32-fold increase (2814 pg/ml to 90434 pg/ml, fig S5c) in expression of IL-8 when stimulated with LPS.

a b c

Figure S5: Expression of IL-8_77_ from cells. Concentration (pg/ml) of total IL-8 (open bars) and IL-8_77_ (shaded bars) from (a) airway epithelial cells and term cord-blood (b) PMNs & (c) monocytes, both unstimulated (U) and when stimulated (S) with IL-1β (airway epithelial cells) or LPS (cord-blood cells). Cell-lines and incubation conditions are represented on the x-axis while concentration (pg/ml) is on the y-axis. Bars are at means (± SEM) of at least three separate experiments.

*Convertase Activity of BALF*

Figure S6 shows a panel of controls where IL-8_77_ (10000 pg/ml = 1.1 nM) was incubated overnight (18 hours) in buffer only (tris buffered saline with 0.5% protease-free BSA), or in the presence of the protease inhibitors AAT (for neutrophil serine proteases; concentration 10 μM), PMSF (for neutrophil serine proteases; concentration 10 mM), 1-10 phenanthroline (for metalloprotenases; concentration 10 mM) and ATIII (for thrombin and plasmin; concentration 150 μg/ml). No significant loss of IL-8_77_ was observed on ELISA after incubating with buffer for 18 hours (mean ± SEM: 9498 ± 720 pg/ml) compared with concentration at 0 hours (9768 ± 356 pg/ml). Comparable concentration of IL-8_77_ was detected after incubation with protease inhibitors only for 18 hours (AAT: 10342 ± 556 pg/ml; PMSF: 9764 ± 432 pg/ml; 1-10 phenanthroline: 9835 ± 536 pg/ml; ATIII: 10786 ± 407 pg/ml). Ethanol was used as a negative control for PMSF and 1-10 phenanthroline, as their diluent in the stock solution; no significant differences were observed in concentration of IL-8_77_ after incubation with ethanol as compared to buffer control (ethanol: 9647 ± 611 pg/ml).

Figure S6: Panel of controls. Concentration of IL-8_77_ detected by ELISA at 0 hours and after incubation in different conditions for 18 hours. Conditions are detailed on the x-axis and concentration of IL-8_77_ (pg/ml) on the y-axis. Open bars represent buffer controls, grey bar represent BALF samples at 0 hour and black bar represents BALF samples after 18 hours incubation (n = 18). All bars are at means (± SEM). Conditions were compared by one-way ANOVA with Dunnets post-hoc test comparing against a control column (BALF at 0 hour). (*** = p < 0.001)

*Conversion of IL-8_77_* *by purified neutrophil proteases at different concentrations*

We first looked at the effect of different concentrations of purified neutrophil proteases on the conversion of rhIL-8_77_. Keeping the molar concentration of IL-8_77_ constant (300 nM), a variable concentration of proteases was added so that the enzyme: substrate ratio ranged between 10:1 (protease concentration 3 μM) to 1:10 (protease concentration 30 nM; a 100-fold change in concentration of proteases). Conversion of IL-8_77_ by the neutrophil serine proteases was found to be protease dose-dependent, with significantly decreased concentration of IL-8_77_ recovered at the higher concentration of proteases (fig S7a-c).

a b

c

Figure S7: Protease dose-response. Conversion of IL-8_77_ by purified human neutrophil (a) elastase, (b) cathepsin-G and (c) proteinase 3 at varying concentration. Ratio of enzyme: substrate is represented on the x-axis while the fold-change of total IL-8 (circles) and IL-8_77_ (squares) from original concentration is represented on the y-axis. Points plotted are means (± SEM) of three independent experiments. Concentration of IL-8_77_ is compared to corresponding concentration of total IL-8 by two-way ANOVA with Bonferroni’s post-test. (* = p < 0.05, ** = p < 0.01, *** = p < 0.001)

*Establishing the neutrophil degranulation assay*

We set up a neutrophil degranulation assay, measuring expression of MMP-9, to test for differences in functional activity between the isoforms of IL-8. Cytochalasin B and fMLP was used as a positive control in these experiments. We compared the amount of MMP-9 produced after degranulation of neutrophils by different concentration (10^-7^ M and 10^-8^ M) of the two standard isoforms of IL-8, rhIL-8_72_ and rhIL-8_77_, in the presence of cytochalasin B. All data was normalised to the MMP-9 expression in the presence of the positive control (cytochalasin B with fMLP) and expressed as a fold-change. As shown in figure S8, significant increase in MMP-9 production was observed with the positive control fMLP (p < 0.001) and in the presence of either of the IL-8 isoforms at a concentration of 10^-7^ M (p < 0.001 for both), compared to expression of MMP-9 in the presence of cytochalasin B only (negative control). No significant differences were noted in the concentration of MMP-9 between the two IL-8 isoforms at this concentration. At a concentration of 10^-8^ M, significant increase in the expression of MMP-9 was noted from the neutrophils by both rhIL-8_72_ (p < 0.001) and rhIL-8_77_ (p < 0.05) compared to negative control. However, expression of MMP-9 was significantly higher when neutrophils were degranulated in the presence of rhIL-8_72_ compared to rhIL-8_77_ (p < 0.001). Thus, at a concentration of 10^-8^ M, the shorter isoform of IL-8 was functionally more potent than IL-8_77_ in this assay. In all future experiments, IL-8 from all conversion experiments were diluted to a final concentration of 10^-8^ M estimated concentration for degranulation of neutrophils.

Figure S8: Establishing the neutrophil degranulation assay. MMP-9 expressed from neutrophils after degranulation by control conditions (open bars), rhIL-8_72_ (grey bars) and rhIL-8_77_ (black bars). Details of conditions are represented on the x-axis while expression of MMP-9 is represented on the y-axis (expressed as a fold change compared to the positive control). Bars are at means (± SEM) of three independent experiments. Difference in means was compared by one-way ANOVA with Tukey’s post-test (comparing all pairs of columns). */*** = significant compared to negative control; ## = significant compared to rhIL-8_72_ at a concentration of 10^-8^ M. (* = p < 0.05, *** = p < 0.001, ## = p < 0.01)

*Functional activity of IL-8 isoforms after processing by proteases*

We then proceeded to test the functional activity of IL-8 isoforms after processing of rhIL-8_77_ by purified neutrophil serine proteases over 24 hours. For these experiments, all samples were diluted to an estimated total IL-8 concentration of 10^-8^ M, the hypothesis being any difference (or change) in functional activity would be due to differences in functional activity between the long and short isoforms. Although NE and CG efficiently converted IL-8_77_, this was not reflected in a change of functional activity (fig S9a-c); no significant differences were observed between functional activity of products from NE or CG and buffer at any time-point. (Functional activity of products after conversion by Pr-3 are discussed in the main manuscript and Figure 5)

a b

c

Figure S9: Functional activity of IL-8 isoforms. Summary of processing of rhIL-8_77_ by (a) buffer, (b) purified elastas and, (c) purified cathepsin G showing concentration of recovered total IL-8 (squares), IL-8_77_ (circles) and MMP-9 from degranulation of neutrophils (triangles) by the products of conversion. Time (hours) is represented on the x-axis while IL-8/MMP-9 (fold change from 0-hour values) is represented on the y-axis. Points plotted are means (± SEM) of three independent experiments. Statistical differences in concentration compared to buffer-control at each time-point was tested by 2-way ANOVA with Bonferroni’s post-test. (*** = p < 0.001).

*Processing of rhIL-8_72_ by Pr-3*

So far, we have shown that processing of rhIL-8_77_ by Pr-3 results in significant reduction of recovery of total IL-8 at 24 hours. To see if this happens with the shorter isoforms of IL-8, we tested rhIL-8_72_ in the same experimental conditions as above. As shown in Figure S10, on incubating rhIL-8_72_ with Pr-3, there was a 20% loss of protein (as detected by IL-8 ELISA) at 4 hours (p<0.001) and 80% loss at 24 hours (p < 0.001).

Figure S10: Processing of rhIL-8_72_ by purified proteinase-3. Recovery of total IL-8 (expressed as a fold change from 0-hour) on incubation with buffer (circles) and proteinase-3 (squares) over 24 hours. Time (hours) is represented on the x-axis while fold-change in concentration (compared to concentration at “0-hour”) is represented on the y-axis. Points plotted are means (± SEM) of three independent experiments. Differences in concentration was tested by two-way ANOVA with Bonferroni’s post-test. (*** = p < 0.001)

**References**

1. de Blic J, Midulla F, Barbato A, Clement A, Dab I, Eber E, Green C, Grigg J, Kotecha S, Kurland G, Pohunek P, Ratjen F, Rossi G. Bronchoalveolar lavage in children. Ers task force on bronchoalveolar lavage in children. European respiratory society. *Eur Respir J* 2000;15:217-231.

2. Nashkevich NN, Akalovich S, Louneva N, Heavner GA, Voitenok NN. A monoclonal antibody and an enzyme immunoassay for human ala-il-8(77). *J Immunol Methods* 2002;270:37-51.

3. Haslett C, Guthrie LA, Kopaniak MM, Johnston RB, Jr., Henson PM. Modulation of multiple neutrophil functions by preparative methods or trace concentrations of bacterial lipopolysaccharide. *The American journal of pathology* 1985;119:101-110.

4. Maheshwari A, Voitenok NN, Akalovich S, Shaik SS, Randolph DA, Sims B, Patel RP, Killingsworth CR, Fallon MB, Ohls RK. Developmental changes in circulating il-8/cxcl8 isoforms in neonates. *Cytokine* 2009;46:12-16.
